# Impact of BMI, osteoporosis, and disc degeneration on post-UBE lumbar stability: a finite element analysis of nonlinear synergistic effects

**DOI:** 10.3389/fbioe.2025.1661626

**Published:** 2025-10-28

**Authors:** Jingbo Ma, Tusheng Li, Rigbat Rozi, Jiaheng Han, Qiang Jiang, Hanshuo Zhang, Xuyan Song, Guotong Zhao, Yu Ding

**Affiliations:** ^1^ Orthopedics of TCM Senior Department, The Sixth Medical Center of People’s Liberation Army General Hospital, Beijing, China; ^2^ Navy Clinical College, Anhui Medical University, Hefei, China; ^3^ Department of Orthopedics, Beijing Chaoyang Hospital, Capital Medical University, Beijing, China; ^4^ Department of Orthopedics, School of Medicine, South China University of Technology, Guangzhou, Guangdong, China

**Keywords:** unilateral biportal endoscopy, finite-element analysis, body mass index, osteoporosis, intervertebraldisc degeneration, lumbar stability

## Abstract

**Objective:**

The aim of this study is to quantify the independent and combined biomechanical effects of increased BMI, osteoporosis, and disc degeneration on lumbar segmental stability after UBE decompression, thereby informing preoperative risk stratification and guiding optimized postoperative rehabilitation protocols.

**Methods:**

A high-fidelity 3D finite-element model of the L3–S1 lumbar spine was developed using CT data of a healthy 31-year-old male volunteer in ANSYS APDL 13.0. This model was used to simulate segmental mechanics after UBE decompression. Four BMI levels (22.86, 26.12, 29.39, 32.65 kg/m^2^), two bone-quality states (normal vs. osteoporotic), and two degeneration grades (mild vs. severe) were configured, resulting in 24 pathological combinations. Axial compressive loads corresponding to each BMI level (457 N, 523 N, 588 N, 653 N) were applied, along with ±10 N·m pure moments. Outcome measures—segmental range of motion (ROM), intradiscal pressure (IDP), and facet-joint von Mises stress—were extracted and validated against published benchmarks to confirm model fidelity.

**Results:**

1. Single-factor effects. With increasing BMI, intradiscal pressure (IDP) at L4–L5 rose by ∼9–12% in non-degenerated discs and loading shifted posteriorly; in degenerated discs, IDP remained lower overall, whereas annular (disc-internal) stress and facet-joint von Mises stress increased. Severe osteoporosis increased vertebral axial-compressive displacement by ∼55% and peak facet-joint stress by ∼48%, indicating reduced structural stiffness and uneven load distribution. Progressive disc degeneration at the index level reduced IDP, most in axial rotation (∼70%), followed by flexion (∼65%) and lateral bending (∼63%), with extension showing the smallest decrease (∼12%); 2. Synergistic effects. Under high BMI (32.65 kg/m^2^) combined with severe osteoporosis and severe degeneration, posterior-element loading increased non-additively: facet-joint von Mises stress rose from 1.02 to 2.47 MPa, exceeding the sum of single-factor effects. Across 24 condition combinations, cranio-caudal load concentration was evident, with disc-internal (annular) stress peaking in the lower lumbar segments (≈1.90 MPa) under high BMI, osteoporosis, and severe degeneration; 3.“Pseudo-stability” window. When severe degeneration coexisted with osteoporosis, axial-rotation ROM at L4–L5 decreased by ∼18% (mechanical locking), yet internal stresses remained high (facet-joint/endplate stresses up to ≈2.5 MPa), indicating that preserved or even reduced gross motion can mask substantial internal overload.

**Conclusion:**

This finite-element study demonstrated that the coexistence of disc degeneration, osteoporosis, and elevated body mass index markedly increases posterior-element loading and disc-internal stresses after unilateral biportal endoscopic decompression. Changes in range of motion were modest overall and tended to decrease when degeneration was combined with osteoporosis, creating a pseudo-stability state in which elevated internal stress is not reflected by gross segmental motion. These findings highlight the importance of considering body weight, bone quality, and disc health together when evaluating postoperative spinal stability and suggest that stress-based assessments may provide a more reliable indicator of hidden instability risk than motion measurements alone.

## 1 Introduction

The incidence of lumbar spinal stenosis (LSS) has been rising annually, severely impairing patients’ health and quality of life. Unilateral biportal endoscopy (UBE) is an emerging minimally invasive technique that has demonstrated significant advantages and broad applicability in managing degenerative spinal disorders (Peng et al., 2025). Compared to conventional open surgeries and single-portal endoscopic procedures, UBE employs two separate portals—one dedicated to visualization and the other to instrumentation—thereby enhancing the intraoperative visual field and surgical maneuverability while minimizing tissue damage and expediting postoperative recovery.

Multiple studies have demonstrated that UBE achieves favorable outcomes in the treatment of lumbar disc herniation and spinal stenosis (Kim et al., 2018). Compared with micro-endoscopic discectomy (MED), UBE delivers superior relief of low back and radicular leg pain and is associated with a significantly lower rate of postoperative complications. While both techniques exhibit comparable operative times and postoperative functional outcomes (e.g., Oswestry Disability Index), UBE demonstrates a clear advantage over MED in reducing postoperative pain scores and accelerating patient recovery (Park et al., 2023). Furthermore, capitalizing on its broader endoscopic view and enhanced instrument maneuverability, UBE is particularly well suited for addressing complex presentations, including bilateral canal stenosis and disc calcification (Ma et al., 2024).

Body mass index (BMI) is a pivotal patient-specific variable in spinal surgery, garnering increasing attention in both clinical and research. Obese patients undergoing lumbar spine surgery face elevated risks of postoperative complications—including infection, poor wound healing, cerebrospinal fluid leakage—and a higher likelihood of reoperation (De la Garza-Ramos et al., 2015). Although it remains debated whether BMI is an independent risk factor, evidence shows that increased BMI correlates with significantly higher rates of complications and reoperation, particularly after lumbar fusion and multilevel procedures (Onyekwelu et al., 2017). Notably, an elevated BMI is associated with suboptimal postoperative outcomes. The biomechanical pathways through which BMI affects spinal stability after UBE remain poorly understood. Clarifying these mechanisms is essential for optimizing patient selection of surgeries and refining surgical techniques to enhance postoperative stability.

From a biomechanical perspective, the excessive load associated with elevated BMI profoundly compromises spinal stability. Under high-load conditions—characterized by diminished spinal stiffness, increased paraspinal muscle activation, and flattening of lumbar lordosis—the spine’s capacity to resist external forces declines sharply when loading reaches 45%–80% of body weight. For instance, Swanenburg et al. demonstrated that resistance to external forces was markedly reduced at 80% body weight (Swanenburg et al., 2020); Häusler et al. observed a substantial loss of stiffness once axial loading exceeded 50% of body weight (Häusler et al., 2020); and Glaus et al. reported significant alterations in dynamic stabilizing mechanisms beyond 45% loading (Glaus et al., 2021). However, studies examining the postoperative biomechanical consequences of elevated BMI and excessive loading remain scarce, underscoring the need for further investigation to inform clinical practice.

LSS predominantly impacts elderly patients, in whom osteoporosis and disc degeneration increase significantly with advancing age and often coexist with elevated BMI. While the majority of existing studies have focused on the impact of individual factors on postoperative recovery or short-term biomechanical alterations, a systematic modeling of segmental stability under the combined pathological conditions of high BMI, osteoporosis, and intervertebral disc degeneration remains lacking. Moreover, the clinical efficacy of UBE is strongly influenced by patient-specific characteristics; in particular, elevated BMI may exacerbate postoperative instability and complication risk, warranting careful preoperative assessment. Osteoporosis reduces the load-bearing capacity of vertebral bodies and discs, increasing intravertebral stress concentration and fracture risk (Chabarova et al., 2024), while disc degeneration further compromises segmental stability, induces abnormal postoperative stress distributions, and accelerates adjacent-segment degeneration (Raftery et al., 2024). These factors likely interact synergistically to exacerbate biomechanical imbalances after UBE, intensifying the load and stress disequilibrium within spinal segments.

Therefore, clarifying the independent and combined effects of elevated BMI, osteoporosis, and intervertebral disc degeneration on post-UBE segmental stability is essential for explaining the underlying biomechanics and for guiding preoperative risk stratification, postoperative rehabilitation, and re-herniation prevention. To this end, we employ a high-fidelity finite-element model to systematically simulate the L3–S1 mechanical response across varied post-UBE conditions, using resection parameters that reflect the actual endoscopic decompression pathway and, to our knowledge, providing the first joint evaluation of BMI, disc degeneration, and osteoporosis after UBE while advancing the clinical concept of “pseudo-stability.” We hypothesize that the combination of high BMI, severe degeneration, and osteoporosis will produce concurrent alterations in L4–L5 ROM with increased IDP and facet-joint von Mises stress relative to lower-risk conditions.

## 2 Methods

### 2.1 Finite element model construction

**FIGURE 1:**
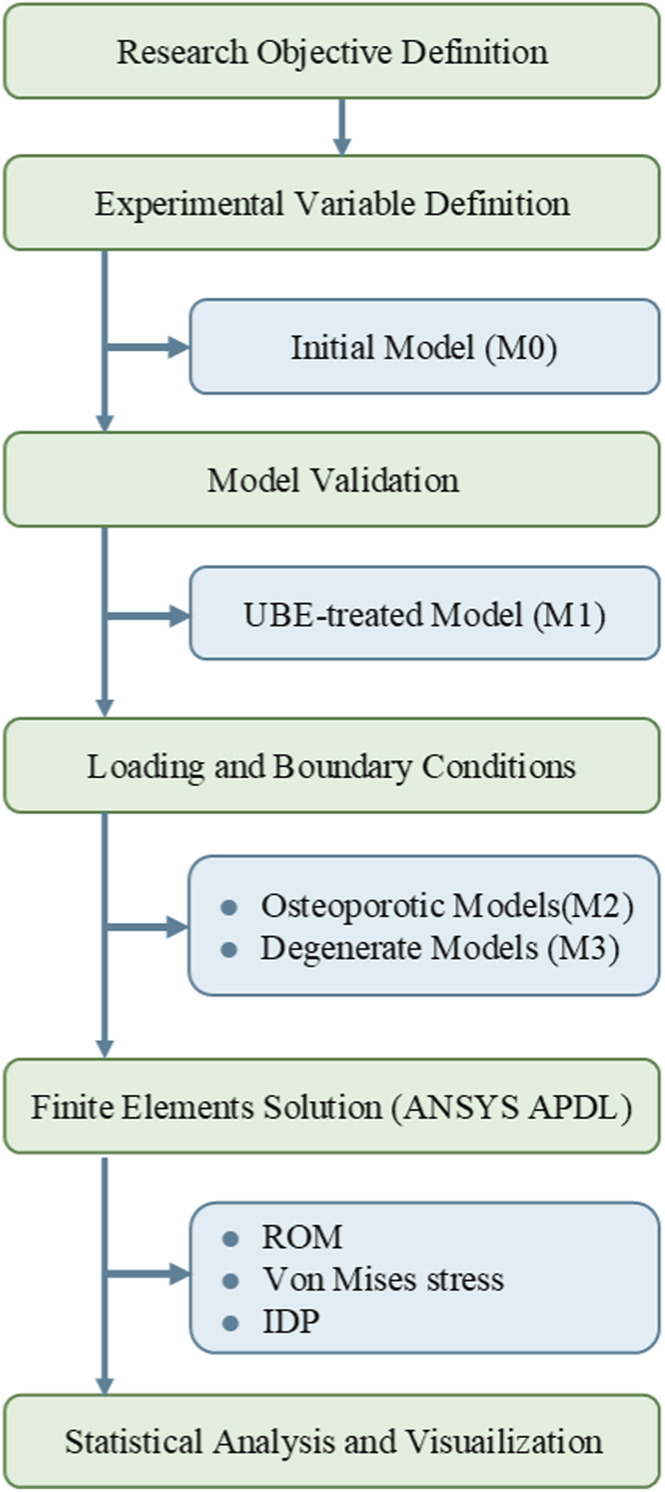
Workflow of finite element modeling and biomechanical analysis of lumbar segments following UBE surgery.

A three-dimensional (3D) finite-element model of the L3–S1 lumbar segment was developed based on imaging data of a healthy 31-year-old male volunteer. This study was approved by the Ethics Committee of the Sixth Medical Center of the General Hospital of the Chinese People’s Liberation Army [Ethics approval No. HZKY-PJ-2025-1]. The model simulated the biomechanical behavior following UBE across various combinations of BMI stratification, osteoporosis, and disc degeneration. Geometry and meshing were performed in ANSYS APDL 13.0 (ANSYS Inc., United States), following the parameterization protocol of Viceconti et al. (2005). The model comprised: Vertebral bodies include cancellous bone, cortical shell, and cartilaginous endplates; Posterior elements include spinous processes, pedicles, transverse processes, and articular facets; Intervertebral discs include annulus fibrosus and nucleus pulposus; Ligaments include anterior longitudinal ligament (ALL), posterior longitudinal ligament (PLL), ligamentum flavum (LF), supraspinous ligament (SSL), interspinous ligament (ISL), intertransverse ligament (ITL), and facet joint capsule (FJC). The overall modeling workflow is summarized in Figure 1.

Material properties for all anatomical components were assigned from published sources (Table 1) (Polikeit et al., 2003; Schweitzer et al., 2007; Bresnahan et al., 2009). The intervertebral disc was modeled as a composite, with annulus fibrosus and nucleus pulposus occupying 56% (674 mm^2^) and 44% (539 mm^2^) of its cross-sectional area, respectively. The annulus fibrosus was modeled as an anisotropic material to reflect its lamellar architecture, while the nucleus pulposus was treated as quasi-incompressible. Facet joints were defined as low-friction sliding contacts (coefficient of friction = 0.1), permitting limited relative motion between articular surfaces. Cartilaginous interfaces were specified as bonded contacts. Ligaments were modeled as nonlinear, tension-only elements using second-order reduced-integration formulations.

**TABLE 1:** Material properties in the present FE models.

Structure	Young’s modulus (MPa)	Poisson’s ratio	Cross-sectional area (mm^2^)	Average length
Vertebrae				
Cortical bone	12,000.0	0.2	-	-
Cancellous bone	340.0	0.2	-	-
Cartilage	10.0	0.4	-	-
Disc				
Endplates	25.0	0.1	-	-
Nucleus pulposus	1.0	0.5	-	-
Annulus fibrosus	500.0	0.5	-	-
Ligaments				
ALL	7.8 (<12.0%)20.0 (>12.0%)	0.4	63.7	20.0
PLL	10.0 (<11.0%)20.0 (>11.0%)	0.3	20.0	12.0
SSL	8.0 (<20.0%)15.0 (>20.0%)	0.3	70.0	22.0
ISL	10.0 (<14.0%)11.6 (>14.0%)	0.3	70.0	13.0
LF	15.8 (<6.2%)19.5 (>6.2%)	0.3	40.0	15.0
TL	10.0 (<18.0%) 58.4 (>18.0%)	0.3	1.8	32.0
CL	7.5 (<25.0%) 32.9 (>25.0%)	0.3	30.0	5.0

ALL, anterior longitudinal ligament; PLL, posterior longitudinal ligament; SSL, supraspinous ligament; ISL, interspinous ligament; LF, ligamentum flavum; TL, transverse ligaments; CL, capsular ligament; FJC, facet joint capsule.

To apply loads and extract responses, remote points were placed on the superior surfaces of the L3–S1 vertebral bodies, facilitating the application of moments and the measurement of outcome metrics such as ROM.

### 2.2 Mesh generation and convergence verification

Mesh generation was conducted in ANSYS Workbench using second-order tetrahedral elements. Element sizes ranging from 1 to 5 mm were tested, and von Mises stress in the L4–L5 nucleus pulposus under a 10 N·m flexion moment was evaluated for mesh sensitivity (Karpiński et al., 2017). Reducing the element size from 2 to 1 mm altered the stress by only 1.45%, satisfying the accuracy criterion; therefore, a 2 mm mesh was adopted for all subsequent simulations. The final mesh comprised 127,622 elements.

Mesh independence was further verified under a combined load of 500 N axial compression and 6 N·m flexion/extension moments (Wilke et al., 1994). Convergence was defined as a change of less than 5% in the target stress between successive refinements, with the relative variation calculated accordingly.

$$\Delta \%=\frac{\left|\sigma_{n}-\sigma_{n}^{-1}\right|}{\sigma_{n}^{-1}}\times 100\;\%$$

**FIGURE 2:**
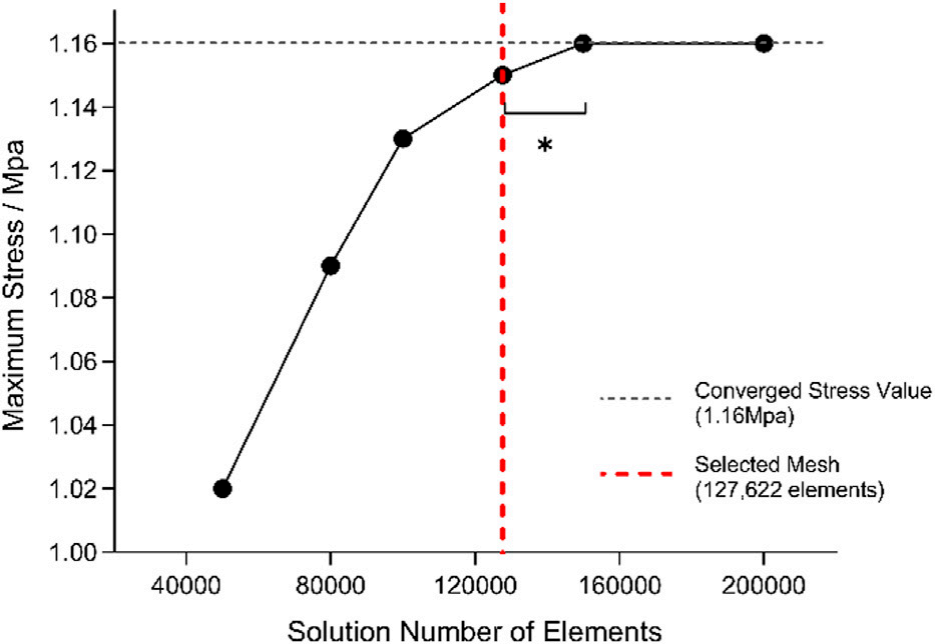
Mesh convergence validation for maximum stress in the finite element model.

Stress values stabilized (variation <5%) once the mesh comprised approximately 1.276 × 10^5^ elements; accordingly, this mesh was chosen for subsequent simulations (Figure 2).

### 2.3 Disc degeneration and osteoporosis modeling

Disc degeneration was simulated according to the Pfirrmann and Thompson grading systems by proportionally reducing the disc height and the area of the nucleus pulposus. Grades I–II were classified as normal, grade III as mild degeneration, and grades IV–V as severe degeneration. Using the validated baseline L3–S1 finite-element model (Zhang and Han, 2023), disc height was reduced by 20% for mild and 60% for severe degeneration; the corresponding loss of nucleus pulposus volume was replaced with annulus fibrosus tissue to conserve total disc volume. Concurrently, the material properties of the endplates and disc tissues were adjusted to reflect endplate sclerosis and other degenerative changes.

Osteoporosis was modeled by reducing bone mineral density (BMD) in both cortical and cancellous bone, thereby reflecting the systemic nature of the disease (Kang et al., 2022). Both bone types were represented as anisotropic materials, with distinct elastic moduli assigned to healthy and osteoporotic conditions. In the osteoporotic model, elastic moduli were uniformly decreased to mirror the reduced stiffness associated with lower BMD. Detailed material properties for both disc-degeneration and osteoporosis scenarios are summarized in Table 2.

**TABLE 2:** Material properties assigned to different tissues in osteoporotic and degenerated disc models.

Structure	Young’s modulus (MPa)	Poisson’s ratio
Mild degeneration group		
Cartilage endplate	24	0.4
Osteophytes	100	0.2
Soft tissue	Hyper-elastic material, C1 = 0.4, C2 = 0.1	
Annulus ground	Hyper-elastic material, C1 = 0.4, C2 = 0.1	
Nucleus pulposus	Hyper-elastic material, C1 = 0.14, C2 = 0.035	
Severe degeneration group		
Cartilage endplate	100	0.4
Osteophytes	100	0.2
Soft tissue	Hyper-elastic material, C1 = 0.9, C2 = 0.23	
Annulus ground	Hyper-elastic material, C1 = 0.9, C2 = 0.23	
Nucleus pulposus	Hyper-elastic material, C1 = 0.19, C2 = 0.045	

### 2.4 UBE surgical model

**FIGURE 3:**
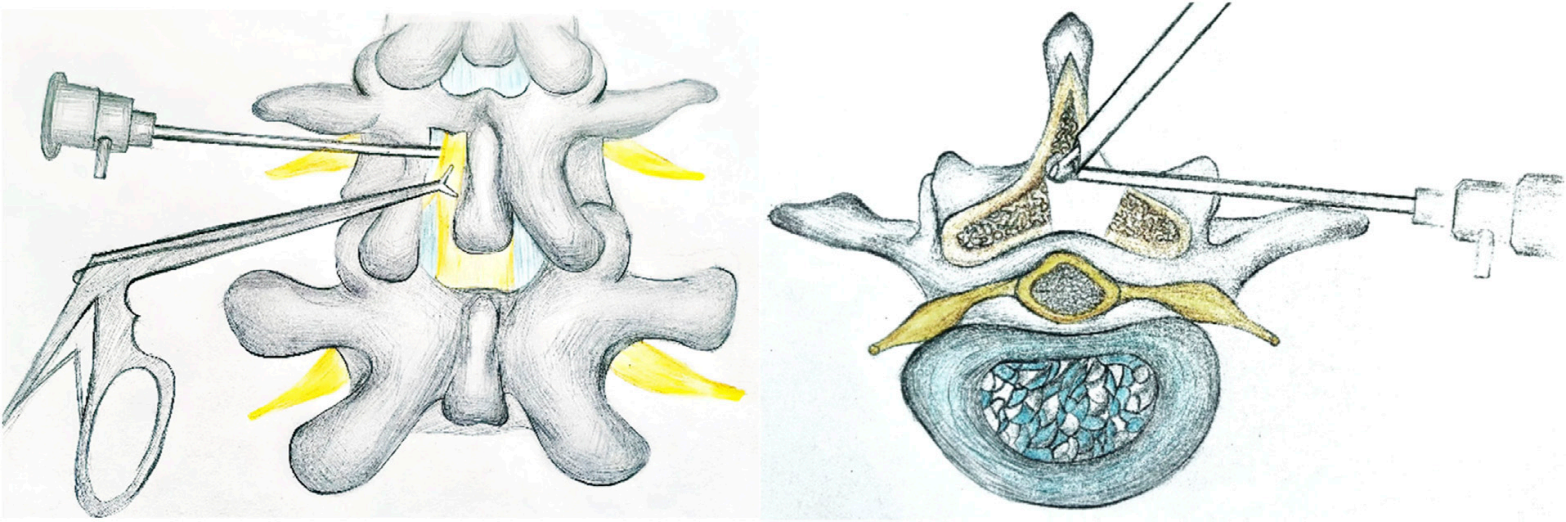
Hand-drawn illustration of the surgical approach in unilateral biportal endoscopy (UBE).

The L4–L5 segment was used to simulate UBE decompression. Through two ipsilateral portals, a 6.9-mm endoscopic trephine created a medial laminotomy with full-thickness flavectomy and limited medial facet trimming to decompress the ipsilateral side, then undercut the spinous base and contralateral lamina to the lateral recess for contralateral decompression; facet cartilage was minimally debrided with subchondral bone preserved, and ≥50% of the bony facet was retained (Sellier et al., 2024). The surgical resections and decompression corridor were parameterized to reflect the clinical pathway and qualitatively checked for anatomical correspondence. The procedural steps and surgical trajectory are depicted in Figure 3.

### 2.5 Loading and boundary conditions

Loading conditions were designed to reflect both BMI-dependent body weight and standardized boundary constraints. Based on a reference height of 1.75 m, four body-mass scenarios (70, 80, 90, and 100 kg) were modeled, corresponding to BMIs of 22.86, 26.12, 29.39, and 32.65 kg/m^2^, respectively. For each case, a vertical compressive load equivalent to two-thirds of body weight (457, 523, 588, and 653 N) was applied to the superior surface of L3 to simulate physiological axial loading (Zhang et al., 2024).

Two distinct load cases were defined and used consistently throughout the study: Scheme Validation: 150 N axial preload applied on L3 and ±10 N·m pure moments to reproduce standard *in-vitro* validation conditions. Scheme Primary simulations: BMI-mapped axial compression (two-thirds body weight; 457/523/588/653 N) applied on L3 and ±10 N·m pure moments; no additional 150 N preload was added. The inferior surface of S1 was fully constrained for both schemes.

The inferior surface of S1 was fully constrained in all degrees of freedom. In Scheme Validation, a 150 N axial preload was applied on L3 prior to the ±10 N·m moments. In Scheme Primary simulations, the BMI-mapped axial compression replaced any additional preload. (Yamamoto et al., 1989; Chen et al., 2001). Pure moments of ±10 N·m were then imposed through remote points to simulate six principal motions: flexion, extension, left and right lateral bending, and left and right axial rotation.

This unified loading scheme ensured consistency across BMI groups and allowed direct comparison of range of motion (ROM), intradiscal pressure (IDP), and facet-joint stress under identical boundary conditions (Karpiński et al., 2019).

### 2.6 Model validation

To ensure predictive stability and biomechanical reliability, we validated the segmental ROM for the intact (M0), osteoporosis (OP), and disc-degeneration (DD) models against established experimental and numerical studies (Cai et al., 2020).

#### 2.6.1 Validation of the intact model (L3–S1)

**FIGURE 4:**
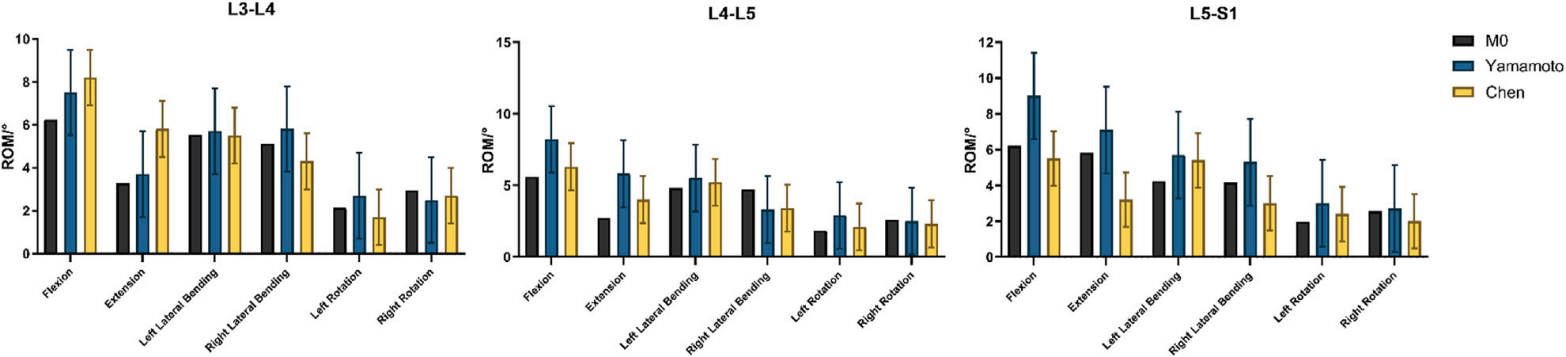
Verification of FE model validity, Comparison of the ROMs with published experimental results.

Under a 150 N axial preload and ±10 N·m pure moments, the ROM of the intact L3–S1 model was extracted for six motion directions: flexion, extension, left and right lateral bending, and left and right axial rotation. Comparison with Yamamoto’s and Chen’s experimental data revealed that all ROM values were within an 8% error margin (Figure 4), thereby confirming the model’s kinematic accuracy and applicability.

#### 2.6.2 Validation of the osteoporosis model

**FIGURE 5:**
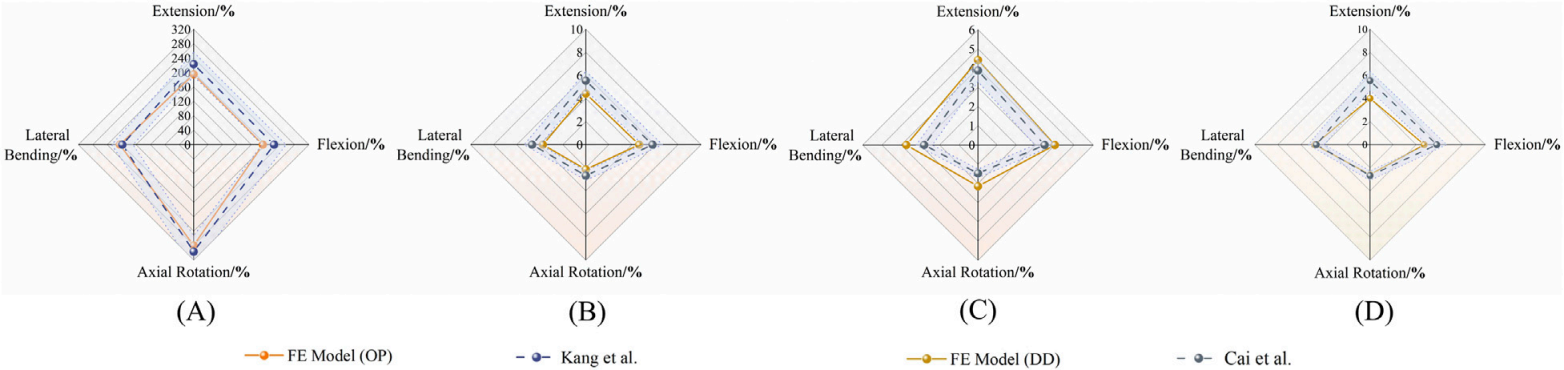
Validation of FE models against published data. Radar plots compare ROM in different motion directions for osteoporotic (OP) and degenerated disc (DD) models. **(A, B)** OP model vs Kang et al. (2022); **(C, D)** DD model vs Cai et al. (2020).

The osteoporosis model simulated severe bone-mass reduction by decreasing the elastic modulus and density of vertebral bone. Under the same loading conditions, overall ROM increased markedly, especially in extension and axial rotation. Comparison with Kang et al.‘s results showed highly concordant trends on the radar plot, confirming that the model accurately reproduces the impact of osteoporosis on segmental stability (Figure 5A). To eliminate dimensional disparities among motion directions and mechanical metrics, all output values were initially converted into relative percentages:

$${\%}_{pathology}=\frac{X_{pathology}}{X_{normal}}\times 100\%$$

Where X_normal_ denotes the value obtained from the intact model (non-degenerated, non-osteoporotic) under identical loading conditions, with the normal condition fixed at 100%.

The deviation between finite-element predictions and literature reference values was quantified using the mean absolute percentage error (MAPE):

$$MAPE=\frac{1}{n}\sum_{i=1}^{n}\left|\frac{X_{FE,i}-X_{lit,i}}{X_{lit,i}}\right|\times 100\%$$

Interlaboratory variation in ROM and IDP for *in vitro* spinal specimens typically falls within ±10%–12% (Wilke et al., 1994; Montanari et al., 2024). Adopting a ±15% tolerance—equivalent to the literature mean ± 1.5 SD (SD ≈ 6–7%) and covering approximately 86% of experimental variability—is both conventional and practical. The 15% threshold also approximates the literature mean ±1.5 × SD (with SD ≈ 6–7% per Heuer.), statistically covering ≈86% of experimental variability. Accordingly, a translucent ±15% band was applied around the literature values on the validation radar plot; finite-element curves confined entirely within this band were deemed to satisfy directional validation.

#### 2.6.3 Validation of the disc-degeneration model

Disc degeneration was modeled in mild (Pfirrmann III) and severe (Pfirrmann IV–V) scenarios by reducing nucleus pulposus modulus and water content, by increasing annulus fibrosus stiffness. Progressive degeneration led to a directional decline in ROM across all motions, with the most pronounced reduction (>70%) occurring in axial rotation for the severe model. Comparison with Cai et al.‘s experimental trends (Figures 5B–D) demonstrated close agreement, confirming the model’s pathological validity.

### 2.7 Outcome measures

**FIGURE 6:**
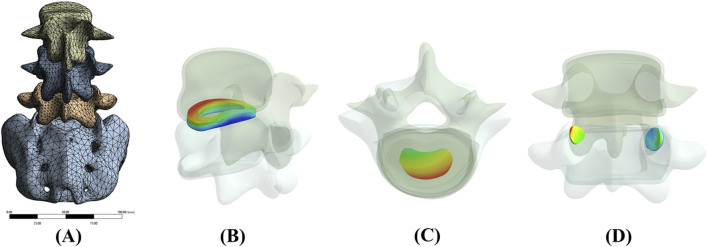
Finite Element Modeling of the Lumbar Spine and Perspective Views of Key Measurement Regions. Notes: **(A)** Full L3–S1 lumbar spine model with refined mesh; **(B)** Stress distribution region in the annulus fibrosus; **(C)** Stress distribution region in the nucleus pulposus; **(D)** Stress distribution region in the Facet joints.

Outcome measures included segmental ROM (°), intradiscal pressure (IDP, MPa) at the nucleus pulposus (pressure surrogate), and tissue von Mises stress (MPa) at the facet joints and endplates Additionally, we evaluated the distribution and magnitude of disc stress-concentration zones for each loading scenario (Figure 6).

## 3 Results

The FE model was first validated against published cadaveric and numerical benchmarks, with all values falling within accepted error margins.

### 3.1 Single-factor effect analysis

The independent biomechanical effects of BMI, osteoporosis, and intervertebral disc degeneration are summarized below.

**FIGURE 7:**
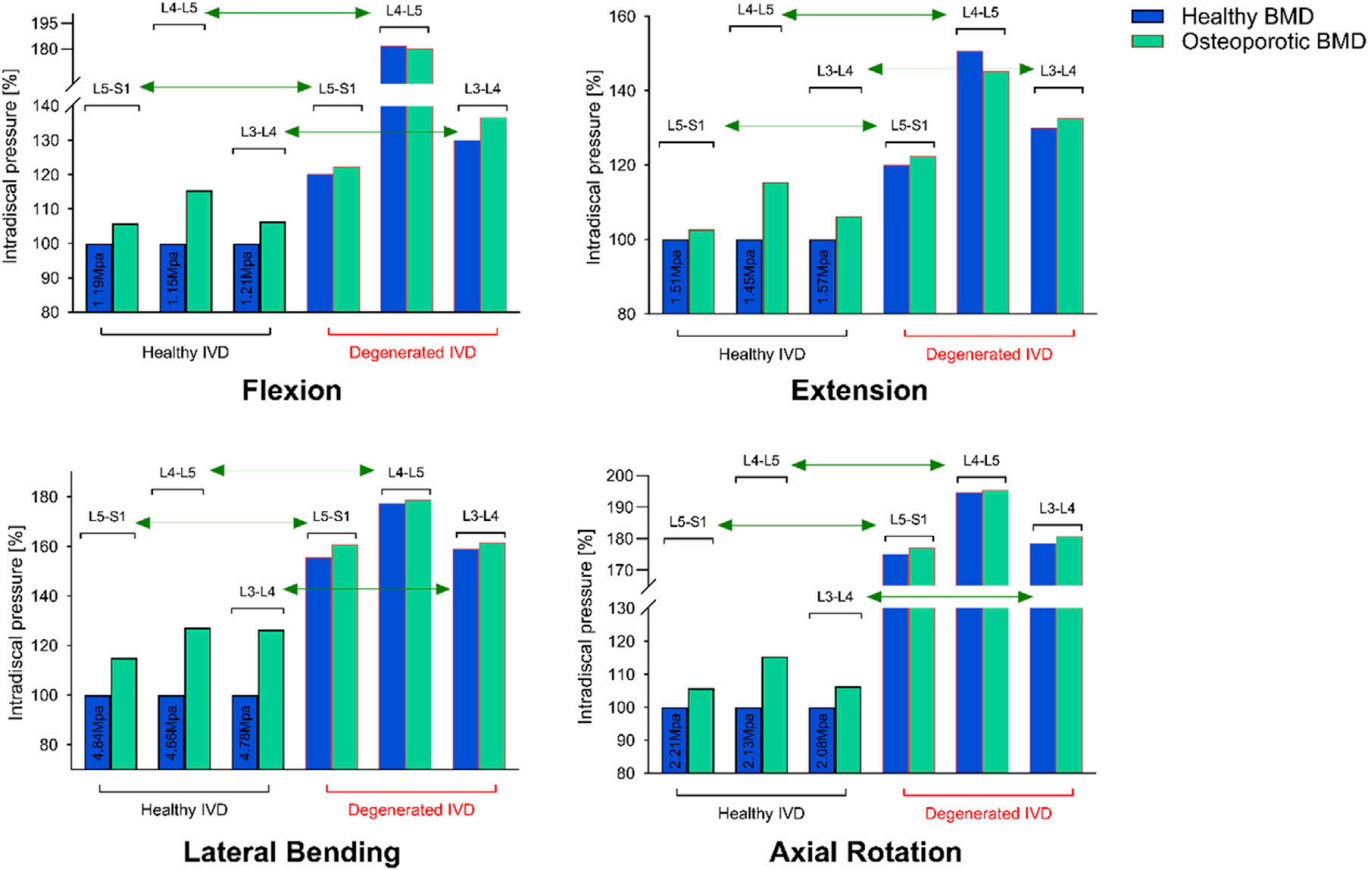
L4–L5 nucleus pulposus pressure (IDP) across degeneration grades and bone densities under four motion states.

1. BMI: Each incremental increase in BMI elevated nucleus pulposus pressure (NP pressure; IDP) by approximately 9%–12% in non-degenerated discs and shifted loads posteriorly; in degenerated discs, NP pressure remained lower overall, whereas annular (disc-internal) stress and facet-joint von Mises stress increased, indicating an amplifying effect of body weight on load transfer.
2. Osteoporosis: Severe osteoporosis increased vertebral axial compressive displacement by approximately 55% and elevated peak facet-joint von Mises stress by about 48%, reflecting reduced structural stiffness and more uneven load distribution.
3. Disc degeneration: Axial rotation was the most sensitive motion, with IDP decreasing by roughly 70% from mild to severe degeneration (Pfirrmann V/Thompson V). Flexion and lateral bending decreased by approximately 65% and 63%, respectively, whereas extension exhibited the smallest decrease (≈12%) (Figure 7).

### 3.2 Analysis of multifactor interactions

**FIGURE 8:**
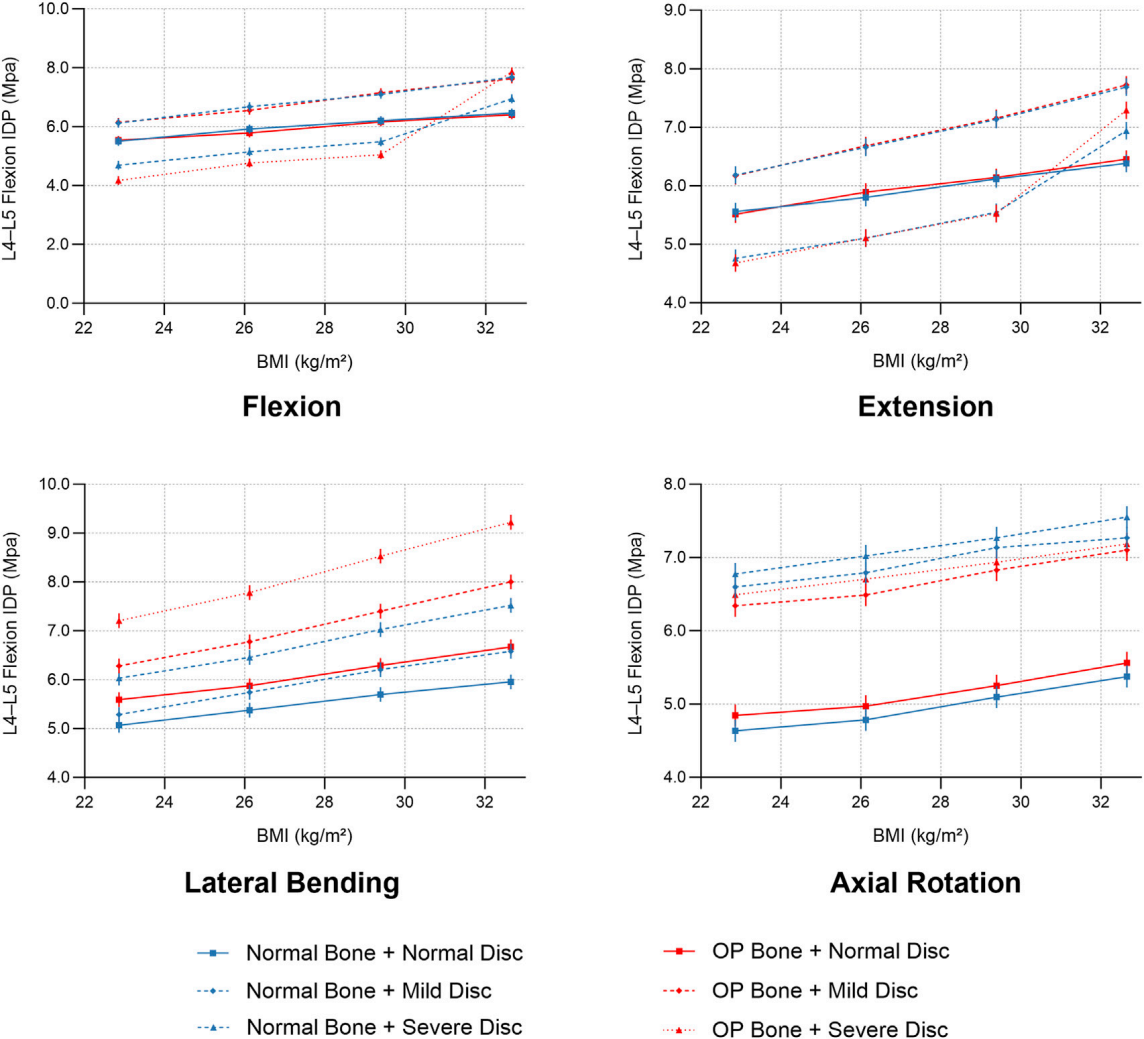
BMI-dependent trends of L4–L5 intradiscal pressure (IDP) under combined conditions of bone density and disc degeneration.

A multifactorial assessment of BMI, osteoporosis, and disc degeneration yielded two primary observations:

1. Nonlinear Synergistic Amplification: Under high BMI (32.65 kg/m^2^) combined with severe osteoporosis and severe degeneration, posterior-element loading increased non-additively. Peak facet-joint von Mises stress rose from 1.02 MPa to 2.47 MPa, exceeding the sum of single-factor effects and indicating pronounced nonlinear amplification.
2. Gradient Changes in Intradiscal Pressure: Across 24 condition combinations (4 BMI levels × 2 BMD categories × 3 degeneration states), a cranio-caudal load concentration was evident: disc-internal (annular) stress increased from L3–L4 to L5–S1 and peaked under BMI ≥29.39 kg/m^2^ with BMD T-score ≤ −2.5 and severe degeneration (up to ≈1.90 MPa in lower segments). (Figure 8).
3. Abnormal Stability Window (pseudo-stability): When severe degeneration coexisted with osteoporosis, relative to the normal baseline, axial-rotation ROM at L4–L5 decreased by ∼18% (same boundary and preload), indicating mechanical locking. Despite reduced mobility, internal stresses remained high (facet-joint and endplate stresses up to ≈2.5 MPa), consistent with a pseudo-stability state in which hidden overload is not captured by gross motion alone.

## 4 Integrated visual analysis of results

**FIGURE 9:**
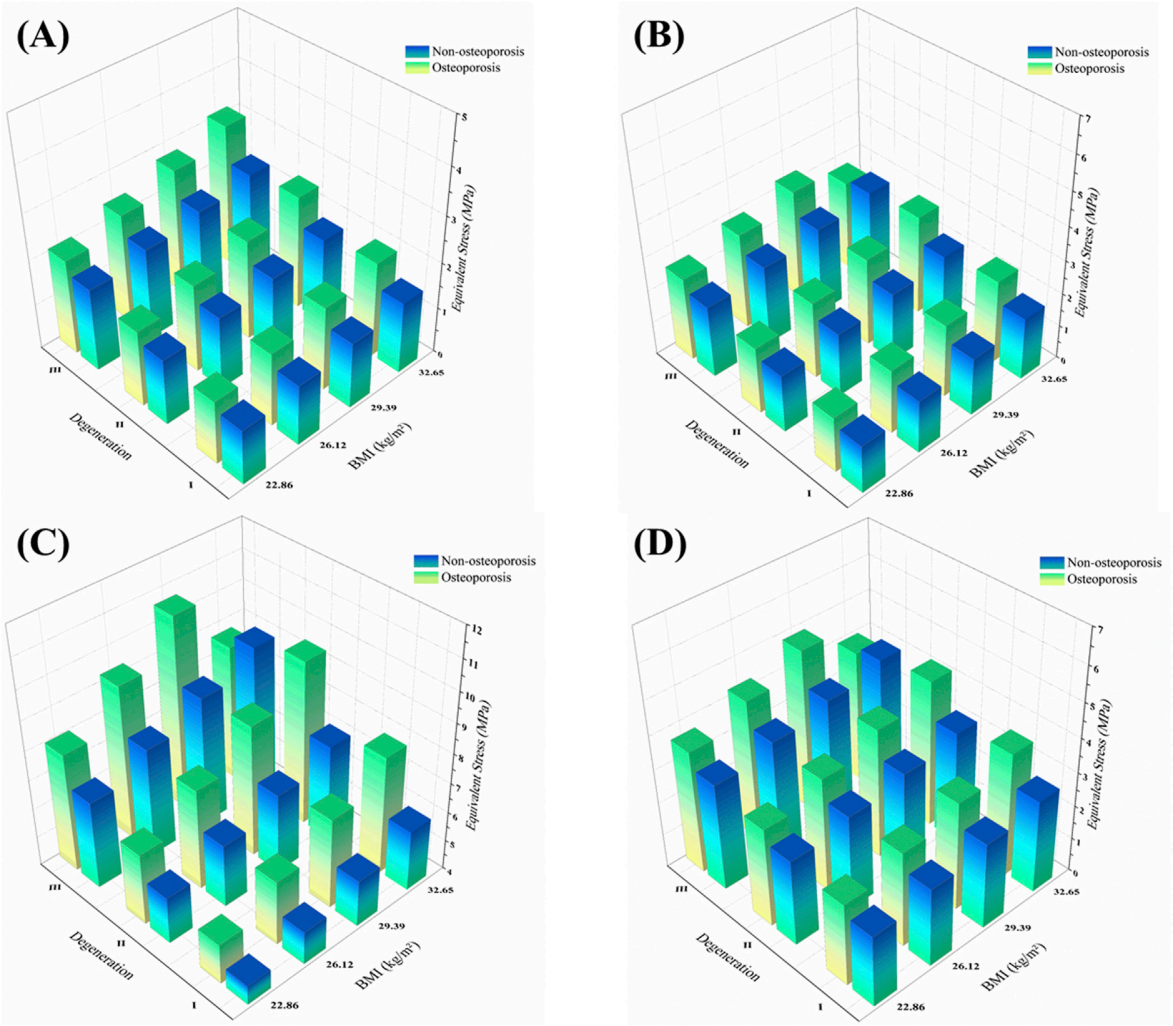
3D distribution of facet joint equivalent stress across BMI, disc degeneration, and osteoporotic status under four motion directions. Notes: **(A)** Flexion; **(B)** Extension; **(C)** Lateral bending; **(D)** Axial rotation.

**FIGURE 10:**
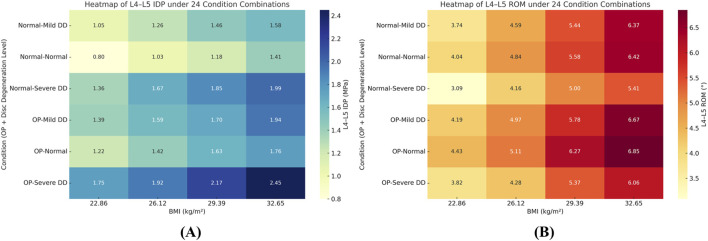
Heatmaps of L4–L5 intradiscal pressure (IDP, MPa) (panel **(A)**) and range of motion (ROM, °) (panel **(B)**) across 24 condition combinations (4 BMI levels × 2 disc-degeneration grades × 3 bone-quality states). Color scale denotes the metric magnitude from low (cool colors) to high (warm colors); numeric ranges are indicated by the colorbar tick labels. Abbreviations: BMI, body mass index; IDP, intradiscal pressure.

**FIGURE 11:**
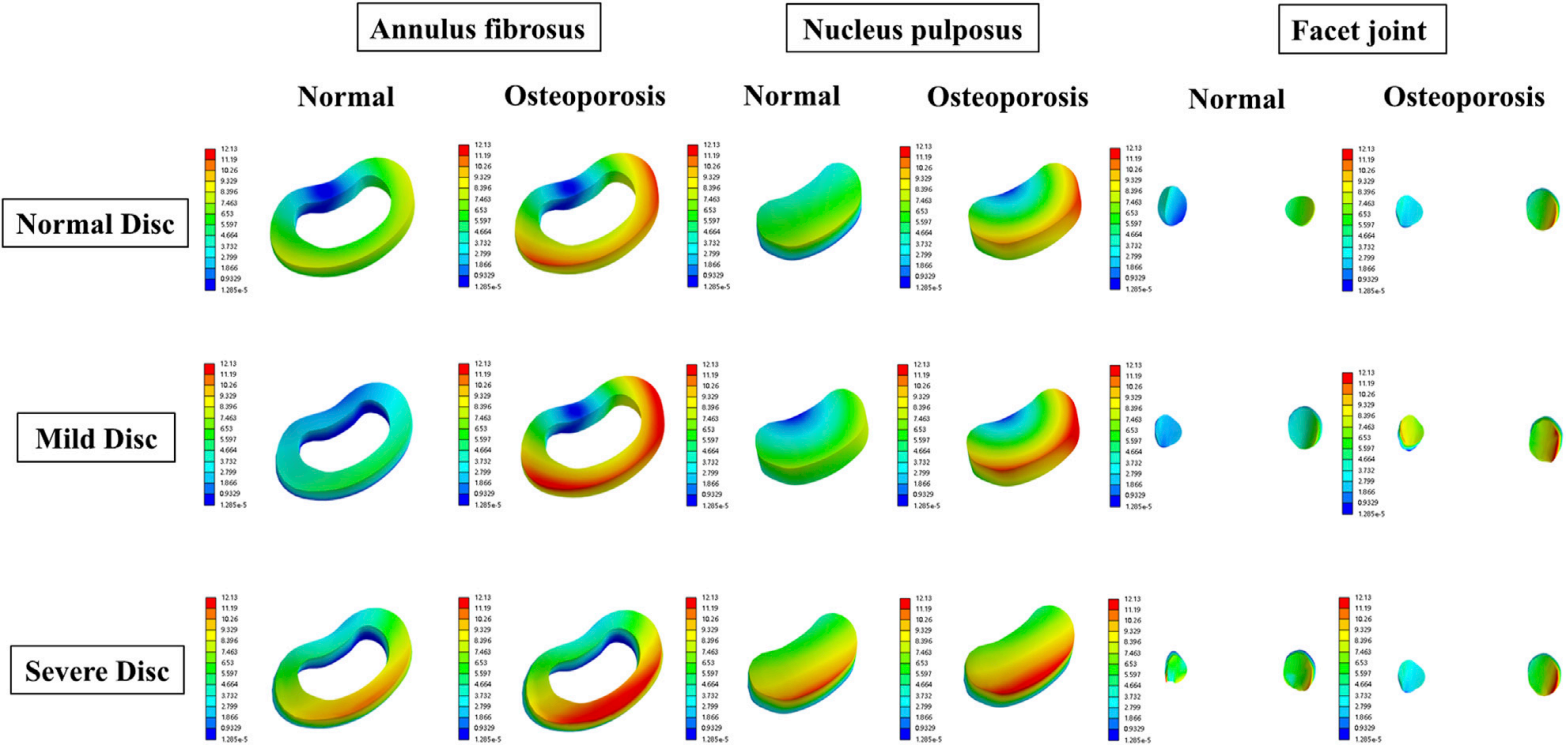
Representative 3D von Mises stress (MPa) distributions in the annulus fibrosus, facet joint, and endplates under BMI = 22.86 kg/m^2^ with different degeneration and bone density states.

**FIGURE 12:**
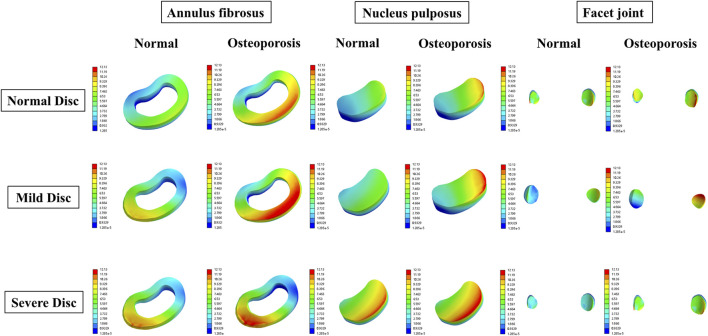
Representative 3D von Mises stress (MPa) distributions in the annulus fibrosus, facet joint, and endplates under BMI = 32.65 kg/m^2^ with different degeneration and bone density states.

To visualize multifactor effects on segmental stability, we synthesized the findings across three core metrics—IDP, segmental ROM, and facet-joint stress (Figures 9–12).

1. IDP and Load Transfer: In non-degenerated discs, IDP increased with BMI and along the cranio-caudal direction; in degenerated discs, IDP remained lower, while annular stress and facet-joint von Mises stress rose—especially under high BMI and osteoporotic bone—highlighting anterior-to-posterior load transfer. Peak disc-internal (annular) stress under combined risk factors reached ≈1.90 MPa in the lower segments (Figure 9).
2. Changes in Segmental Range of Motion and Stability: ROM generally decreased with degeneration at the index level; BMI produced modest increases in certain motions under the pure-moment setup, whereas osteoporosis alone had limited impact on ROM. Notably, in the severe degeneration + osteoporosis setting, axial-rotation ROM decreased by ∼18% (mechanical locking), reinforcing the pseudo-stability pattern in which internal stress escalation rather than gross mobility reflects the true instability risk (Figure 10).
3. Facet-Joint and Endplate Stress-Concentration Characteristics: Under high BMI + severe degeneration + osteoporosis, facet-joint von Mises stress concentrated at the posteromedial L4–L5 facets and central endplate, with peaks rising from 1.02 MPa to 2.47 MPa; stresses escalated nonlinearly in axial rotation and flexion across pathological combinations—consistent with posterior-element overload as a potential mechanism for postoperative pain and secondary degeneration (Figures 11, 12). To facilitate an integrated understanding of the main biomechanical outcomes, a synthetic summary table was compiled. Table 3 presents the key changes in ROM, intradiscal pressure (IDP), annular stress, and facet-joint stress under the most representative single- and multi-factor conditions.

**TABLE 3:** Synthetic summary of key biomechanical outcomes at L4–L5 under critical single- and multi-factor conditions.

Condition	ROM (index level)	IDP (NP pressure)	Annular (disc-internal) stress	Facet-joint stress	Clinical takeaway
BMI +1 grade; non-degenerated disc	Small increase in some motions	↑ about 9%–12%	≈ baseline to slight ↑	≈ baseline to slight ↑	Higher body weight raises anterior load and begins shifting load posteriorly
BMI +1 grade; degenerated disc	Minor change overall	Low overall (degeneration-related)	↑	↑	Weight amplifies posterior load transfer in a compromised disc
Severe osteoporosis vs. normal BMD	Little change to slight decrease	—	Local stress concentration ↑	↑ about 48%	Reduced bony stiffness and uneven load sharing
Severe degeneration vs. mild	↓ in all motions; largest in axial rotation	↓ ∼70% in rotation; ↓ ∼65% flexion; ↓ ∼63% lateral bend; ↓ ∼12% extension	↑	↑	Degeneration lowers NP pressurization and shifts load to annulus and facets
Severe degeneration + osteoporosis	↓ axial-rotation ROM by ∼18% (mechanical locking)	Low	High; persistent	Up to ∼2.5 MPa	Pseudo-stability: reduced motion with sustained internal overload
High BMI 32.65 + severe osteoporosis + severe degeneration	Modest overall change	Low	Peak ≈1.90 MPa in lower segments	1.02 → 2.47 MPa (non-additive increase)	Nonlinear amplification of posterior-element loading; highest risk scenario

Effects reported relative to the baseline model (normal BMD, non-degenerated disc, lower BMI). Loads and constraints are identical across comparisons. IDP, denotes nucleus pulposus pressure; annular stress denotes disc-internal solid stress.

## 5 Discussion

### 5.1 Alterations in load-transfer mechanisms after UBE

Decompressive UBE removes portions of the lamina and ligamentum flavum, reducing posterior-column bending stiffness and shifting greater moments to remaining structures. Our simulations confirmed a decrease in flexion-extension stiffness of lumbar segments following decompression. Montanari et al. similarly reported that two-level laminectomy *in vitro* markedly increased lumbar flexion ROM and disc strain, as loss of posterior support forced anterior discs and posterior facets to bear higher bending loads (Montanari et al., 2024). An elevated BMI further exacerbates this load transfer: increased body weight raises axial spinal loads, thereby imposing additional stress onto the anterior column. Takatalo et al. demonstrated in a finite-element study that obesity significantly increased lumbar segmental mobility and intradiscal pressure compared to normal weight (Takatalo et al., 2013). Epidemiological data link higher BMI to accelerated degeneration of discs and facets, thereby increasing lumbar stenosis and spondylolisthesis risk (Schuller et al., 2011). Schuller, Charles, and Steib et al. found that 71.4% of patients with L4–L5 degenerative spondylolisthesis were overweight (BMI >25), attributing slips in part to obesity-induced overload (Alexiou and Voulgaris, 2013). Consistent with these findings, our model demonstrated that under high BMI, bending loads after UBE are predominantly borne by the anterior column and facet joints, underscoring the amplifying effect of obesity on postoperative load redistribution.

### 5.2 Independent effects of three pathological factors on postoperative segmental stability

#### 5.2.1 High BMI: increased axial load and flexion–extension mobility

This study first elucidated the individual biomechanical impact of elevated BMI on the L4–L5 segment following UBE. Increasing BMI produced a linear rise in a postoperative segmental ROM and IDP. Specifically, flexion-extension ROM at L4–L5 was approximately 35% greater at a BMI of 32.65 kg/m^2^ than at a normal BMI. These findings align with Singh, who reported a 22%–33% increase in lumbar functional–unit ROM and concurrent elevations in disc stress and deformation when BMI increased from normal to obese (Singh et al., 2024). Similarly, Zhang et al. demonstrated that higher BMI markedly augmented equivalent stress and maximum deformation of the L4–L5 nucleus pulposus, particularly under flexion. Collectively, these results support the mechanistic principle that “greater body mass equates to greater load”: elevated BMI increases axial forces and bending moments on the disc, leading to greater postoperative segmental mobility and IDP (Samartzis et al., 2011). Clinically, this loading effect corresponds with higher rates of disc degeneration and low-back pain in obese patients and helps explain why BMI is a major risk factor for postoperative disc recurrent herniation after endoscopic surgery. Accordingly, weight management is essential for maintaining postoperative segmental stability and limit disc stress to a safe range (Zou et al., 2024). Elevated BMI increases baseline axial compression and amplifies bending/rotational moments, intensifying coupling between shear and torsion at the motion segment. In discs with reduced NP pressurization, this additional load preferentially shifts bearing from the anterior column to posterior elements, engaging the annulus and facet joint more heavily. Clinically, higher BMI warrants stricter spine-neutral hygiene, short-term bracing as needed, and delayed high-load/rotational tasks alongside sustained weight reduction.

#### 5.2.2 Osteoporosis: weakened support and amplified vertebral strain

Osteoporosis—characterized by reduced bone mineral density (BMD)—undermines postoperative segmental stability. In our simulations, a 30% reduction in BMD increased anterior compressive strain of the L4 vertebral body by 28% and elevated L4–L5 flexion ROM, indicating that diminished osseous support renders the segment more deformable. Kang similarly reported that under identical physiological loads, disc stresses in osteoporotic lumbar segments exceeded those in normal-BMD spines, with the greatest elevations at L4–L5; nucleus pulposus stress rose significantly in all motion directions, concentrating in the inferior endplate and cancellous bone. Zou et al. confirmed that post-UBE ROM and disc stress were higher in osteoporotic models compared to normal-bone models under identical decompression conditions (Zou et al., 2024). Chabarova et al. further demonstrated that osteoporosis can increase disc deformation by over 30% and reduce overall spinal load-bearing capacity by approximately 30% (Chabarova et al., 2024). Together, these findings indicate that reduced vertebral stiffness forces the disc to accommodate greater deformation and stress, increasing segmental compliance (ROM) and intensifying internal stress concentrations. Consequently, even with unchanged surgical fixation, postoperative osteoporosis diminishes internal stability and alters stress distribution, predisposing the segment to cumulative microdamage and amplified deformation.

#### 5.2.3 Disc degeneration: inducing “rotational instability” and reconstructing motion patterns

Severe disc degeneration substantially alters postoperative segmental kinematics. In our simulations, axial-rotation ROM at L4–L5 increased by approximately 70%, and flexion–extension ROM exhibited asymmetry, with hyper-flexion and restricted extension. This aberrant mobility arises from degenerated disc mechanics: the reduced nucleus pulposus hydration and elasticity, annular fiber rupture and laxity, and diminished rotational damping grant the segment greater rotational freedom. Ibarz et al. similarly demonstrated that progressive disc deterioration increases lumbar ROM and provokes hypermobility and instability (Jentzsch et al., 2015). Clinically, the “instability-phase” hypothesis posits an initial hypermobile stage—especially in rotation and lateral bending—followed by stiff stabilization in advanced degeneration (Kirkaldy-Willis theory).

### 5.3 Nonlinear biomechanical responses under synergistic multifactor interactions

#### 5.3.1 High BMI plus osteoporosis: posterior-column stress concentration

Combining high BMI with osteoporosis revealed pronounced coupling effects on posterior-column biomechanics. In our model, this dual pathology increased L4–L5 facet-joint stress per unit area by 42%, despite unchanged articular contact area. Disc-height loss and postural alterations are likely to precipitate earlier facet engagement, so the redistributed load elevates pressure on each cartilage unit, aggravating stress concentration. Mechanistically, obesity-induced axial load exceeds the bearing capacity of osteoporotic vertebrae, shifting load to posterior facets and capsular structures and elevating articular pressures abnormally. This inference aligns with epidemiological evidence linking obesity to zygapophyseal osteoarthritis, suggesting that excessive body weight accelerates wear and degeneration of facet-cartilage (Bashkuev et al., 2018). Our findings provide biomechanical support for the notion that: when high loads meet low bone strength, the load path shifts posteriorly, imposing excessive stresses on facet joints and potentially provoking degeneration and pain. This interaction exemplifies a nonlinear superposition: osteoporosis amplifies obesity-driven stress redistribution, and high BMI intensifies facet overload in weakened bone. Accordingly, postoperative rehabilitation and follow-up should monitor posterior-column joint loads in obese, osteoporotic patients to prevent occult facet degeneration.

#### 5.3.2 Triple-factor superimposition: limited stiffness reduction with marked internal stress rise

When BMI elevation, osteoporosis, and disc degeneration co-occur, external mechanical responses change only modestly, yet internal stresses escalate dramatically. Under this triad, peak IDP rose by 54% compared to the high-BMI–only scenario, and local shear strain and stress in the annulus fibrosus also increased significantly. Thus, the segment enters a “pseudo-stability” state—apparent stiffness is preserved while internal stress overload mounts. Disc degeneration induces height loss and annular fibrosis, which can stiffen the segment in certain directions, thereby potentially obscuring detectable increases in ROM. However, high axial loads transmit directly through the sclerotic disc, accumulating abnormal internal stresses. Concurrently, osteoporosis reduces endplate buffering capacity, disrupting uniform pressure distribution and forcing concentrated loads onto the nucleus pulposus. Consequently, surface motion amplitude underrepresents internal tissue stress severity. Although little research has examined all three factors simultaneously, this parallels the “restabilization” phase in advanced degeneration, where osteophyte formation restores external stability while internal discs endure high stress and adjacent segments bear increased loads (Zahari et al., 2017). Our findings clarify the biomechanical basis of postoperative pseudo-stability in high-risk patients: preserved or reduced segmental motion on imaging may conceal internal overload and damage. Clinicians should therefore remain vigilant for latent injury when multiple adverse factors coincide.

### 5.4 Study novelty and comparison with previous literature

Prior biomechanical studies have predominantly employed univariate designs, examining obesity, osteoporosis, or disc degeneration in isolation. Obesity is a well-established risk factor that increases lumbar loading, elevates IDP, and accelerates degeneration. Similarly, osteoporosis characterized by reduced bone mineral density—weakens spinal load-bearing capacity, yielding greater displacements and stress responses under equivalent loading or surgical conditions (Zou et al., 2024). Han et al. used finite element analysis to compare disc degeneration in normal versus osteoporotic spines, demonstrating that degeneration in osteoporotic models redistributes loads—decreasing stresses in cortical bone and endplates while increasing stresses in cancellous bone and posterior facets. Cai et al. simulated graded L4–L5 degeneration, finding that severe degeneration reduced segmental ROM by up to ≈75% (e.g., axial rotation) while increasing adjacent-segment ROM, IDP, annulus-fibrosus stress (0.4–2.6 MPa), and neighboring facet-joint loads. Although these single-factor investigations clarified individual influences, they failed to account for non-linear superposition when pathological factors coexist. In contrast, our study represents the first attempt to incorporate elevated BMI, osteoporosis, and disc degeneration within a unified finite-element framework, thereby capturing their synergistic effects on L4–L5 biomechanics and providing a more clinically representative model.

Besides, most prior studies have not incorporated the biomechanical impact of the surgical pathway itself. Most finite element and *in vitro* investigations simulate the intact preoperative spine, with little consideration of decompressive alterations. For example, stand-alone laminectomy often compromises segmental stability and can precipitate vertebral slippage (Simon et al., 2022), whereas tubular minimally invasive decompression preserves portions of the posterior column—maintaining global stability but subjecting remaining structures to elevated local stresses (Dhar et al., 2024). In a recent finite-element comparison of percutaneous transforaminal endoscopic discectomy (PTED) versus unilateral biportal endoscopy (UBE) at L4–L5, Zou, et al. reported that both techniques minimally affected overall ROM and disc stress, with UBE exhibiting local stability loss only under specific motions (e.g., L4–L5 maximal displacement rose by ≈ 16–18% during lateral bending and peak stress increased by ≈ 11.7% in left axial rotation). Overall, biomechanical models that explicitly simulate the surgical pathway remain scarce.

By integrating three common clinical pathologies—elevated BMI, osteoporosis, and disc degeneration—into a finite-element model that includes postoperative decompressive anatomy, this study systematically deciphers the layered mechanisms governing L4–L5 stability and load response after UBE. Our results validate the Introduction’s hypothesis that the coexistence of these factors induces stress imbalance and latent instability after UBE. Individually, each pathology alters segmental mobility and internal stress; in combination, they interact nonlinearly, creating a “pseudo-stability” that conventional assessments may overlook. These findings underscore the need for comprehensive preoperative evaluation and postoperative management that account for multiple risk factors, distinguishing apparent stability from internal stress overload. This paradigm offers a theoretical foundation for personalized rehabilitation, re-herniation prevention, and individualized patient care. This work co-frames BMI, degeneration, and osteoporosis in a unified, procedure-concordant FE model to minimize cross-study heterogeneity, and delivers practical guidance for BMI ≥30 with low bone mass and advanced degeneration—namely avoid early end-range axial rotation/lateral bending, implement time-bound weight control, and initiate/schedule anti-osteoporotic therapy.

### 5.5 Clinical implications and translational relevance

In light of our findings, several clinical implications emerge. First, patients with high BMI, osteoporosis, or severe disc degeneration demonstrated a greater tendency toward postoperative instability after UBE. This suggests that these risk factors should be carefully evaluated in preoperative planning, and whenever possible, optimized through weight reduction and osteoporosis management prior to surgery. Second, the observed “pseudo-stability” phenomenon indicates that conventional kinematic assessments may underestimate hidden stress overload, particularly in obese or osteoporotic patients with advanced degeneration. Clinicians should therefore exercise caution when interpreting preserved or mildly reduced ROM on imaging, as this may mask substantial internal biomechanical risks. Third, our results highlight the importance of tailored postoperative rehabilitation. Specifically, strategies such as core muscle strengthening, sustained weight control, and pharmacological or lifestyle interventions to improve bone density may help mitigate stress overload, prevent recurrent instability, and reduce the likelihood of adjacent-segment degeneration.

### 5.6 Limitations and future directions

Although finite-element analysis has been widely used in spinal biomechanics since the 1970s and offers low susceptibility to external variables with strong controllability, its fundamentally static nature cannot fully replicate complex neuromuscular control (Ribeiro et al., 2024). Our simulations were performed under quasi-static loads (BMI-matched axial compression plus ±10 N·m pure moments) to provide stable, comparable end-range responses across 24 combinations; however, this design does not capture time-dependent tissue behavior, muscle activation patterns, or repetitive daily activities. Moreover, the current model is based on the lumbar geometry of a single healthy male, limiting generalizability across sex, body habitus, and age. Consequently, the absolute magnitudes of ROM, IDP, and facet stress should be interpreted with caution and not read as task-specific values (e.g., walking, lifting) nor as implying fatigue or cumulative damage; the intended interpretation is trend-level—under the modeled conditions, higher BMI and the presence of osteoporosis or severe degeneration are associated with reduced post-UBE segmental stability and increased local tissue loading. To broaden realism and quantify variability, future work will (i) generate multi-subject models from CT datasets spanning age/sex morphologies, (ii) perform probabilistic/parametric analyses that vary key geometric and material parameters within reported ranges, (iii) couple musculoskeletal estimates of muscle forces to subject-specific anatomies, and (iv) incorporate fatigue behavior with representative cyclic load histories (e.g., repeated flexion–extension and axial-rotation, and a walking-like pattern). Finally, validation against longitudinal clinical outcomes will be crucial to develop individualized, dynamic biomechanical assessments.

## 6 Conclusion

Using a validated high-fidelity L3–S1 finite-element model, this study systematically examined lumbar segmental biomechanics after unilateral biportal endoscopic decompression under the combined influence of body mass index, osteoporosis, and disc degeneration. The results showed that disc degeneration and osteoporotic bone loss reduced intradiscal pressure and segmental range of motion while shifting loads to the annulus and posterior elements, thereby increasing facet and endplate stresses. Elevated body mass index further amplified these load-transfer effects, with posterior-element stresses showing the most pronounced increases when all three factors coincided. Importantly, under severe degeneration combined with osteoporosis, segmental motion decreased while internal stresses remained persistently elevated, representing a pseudo-stability state. Clinically, these findings highlight the need for comprehensive postoperative management strategies, including weight control, bone quality optimization, and close monitoring of degenerative progression, to prevent hidden instability and improve surgical outcomes.

## Data Availability

The raw data supporting the conclusions of this article will be made available by the authors, without undue reservation.

## Abbreviations

UBE, unilateral biportal endoscopy; LSS, lumbar spinal stenosis; FE/FEA, finite-element/finite-element analysis; BMI, body mass index; ROM, range of motion; IDP, intradiscal pressure; NP, nucleus pulposus; ALL/PLL, anterior/posterior longitudinal ligament; LF, ligamentum flavum; SSL, supraspinous ligament; ISL, interspinous ligament; ITL, intertransverse ligament; FJC, facet joint capsule.
